# Connections Matter: Social Networks and Lifespan Health in Primate Translational Models

**DOI:** 10.3389/fpsyg.2016.00433

**Published:** 2016-04-22

**Authors:** Brenda McCowan, Brianne Beisner, Eliza Bliss-Moreau, Jessica Vandeleest, Jian Jin, Darcy Hannibal, Fushing Hsieh

**Affiliations:** ^1^Department of Population Health and Reproduction, School of Veterinary Medicine, University of California, DavisDavis, CA, USA; ^2^California National Primate Research Center, University of California, DavisDavis, CA, USA; ^3^Department of Psychiatry and Behavioral Sciences, University of California, DavisDavis, CA, USA; ^4^Department of Statistics, University of California, DavisDavis, CA, USA

**Keywords:** translational, nonhuman primates, health status, well-being, social network analysis

## Abstract

Humans live in societies full of rich and complex relationships that influence health. The ability to improve human health requires a detailed understanding of the complex interplay of biological systems that contribute to disease processes, including the mechanisms underlying the influence of social contexts on these biological systems. A longitudinal computational systems science approach provides methods uniquely suited to elucidate the mechanisms by which social systems influence health and well-being by investigating how they modulate the interplay among biological systems across the lifespan. In the present report, we argue that nonhuman primate social systems are sufficiently complex to serve as model systems allowing for the development and refinement of both analytical and theoretical frameworks linking social life to health. Ultimately, developing systems science frameworks in nonhuman primate models will speed discovery of the mechanisms that subserve the relationship between social life and human health.

## Introduction

No human exists in a social vacuum-rather, we live in societies full of rich, complex relationships that influence both our physical and mental health. The ability to treat and prevent illness and improve human health requires not only a detailed understanding of the complex interplay of biological systems contributing to disease processes but also how social contexts influence such biological systems. Approaches that empirically recognize the inherent complexity of the effects of social life on health are critical and must be applied across the lifespan. Health is an emergent phenomena which arises from the interplay between complex systems that are themselves influenced by a myriad of factors. These factors are specific to the individual (e.g., personality or temperament, genetic predispositions, ancestry) and/or specific to the environment (e.g., different types of environmental variables; social stressors). A longitudinal systems science approach provides methods uniquely suited to elucidate the mechanisms by which social systems influence health by investigating the effect of social systems on the interplay between biological systems (e.g., immune system, neuroendocrine system) across the life span. Here, we describe one possible systems science approach as applied to rhesus macaques (*Macaca mulatta*), a nonhuman primate species sharing close evolutionary history and behavioral biology with humans. We argue that despite the promise of new systems science approaches, many of those approaches have been developed without social and biological data in mind, necessitating their refinement in the context of complex biological and social systems—rather than, for example, a complex physical system or simulated system on which many new approaches are developed. Nonhuman primate model systems, such as those of rhesus macaques, are sufficiently complex to allow modeling of both biological and social systems while being tractable enough to collect nuanced and detailed data that is ultimately required for the refinement of systems science approaches.

## The relationship between social environment and health

Social life and its interaction with factors related to the individual influence physical and mental well-being in both humans and nonhuman primates (Walker et al., 1999; Vandeleest et al., 2013). However, the multi-scale dynamic nature of the effect of social relationships on health remains poorly understood. One reason for this is that extant studies have computational obstacles that limit study designs to either specific individual attributes (e.g., gender, age) or specific physiological system dynamics [e.g., hypothalamic-pituitary-adrenal (HPA) axis or immune system] in isolation. That is, simply because of limitations in experimental design and data analysis, we have yet to be able to effectively model the extraordinarily complex dynamic nature of the social environment in concert with a full picture of what it means for an individual to “be healthy.”

Decades of research have documented the effect of social context on physical and mental health in humans and nonhuman primates. In humans, characteristics of the social environment such as socioeconomic status influence diverse health outcomes ranging from cardiovascular disease (e.g., Winkleby et al., 1992) to mood disorders such as depression (e.g., Gilman et al., 2002) to mortality (e.g., Blaxter, 1987). For example, long-term studies of British civil service workers have provided decades of data demonstrating that social status has an important impact on a wide variety of outcomes such as cardiac (e.g., angina, ischaemia) and respiratory (e.g., bronchitis) health (Marmot et al., 1991; Singh-Manoux et al., 2003)—the lower a person's status the greater prevalence of disease. Low social status is associated with unpredictability of income, housing, healthcare access, as well as low control over working conditions, all features that contribute to chronic stress and illness (Weissman et al., 1991; Väänänen et al., 2008; Kim et al., 2012). Similarly, as discussed below, research from our group (and others) demonstrates that nonhuman primate's absolute social rank (akin to human class) and the certainty of that social rank (akin to predictably/unpredictably) are both key factors influencing individual-level health outcomes (Vandeleest et al., in revision).

The number and quality of specific social relationships are an additional aspect of social life which influence health has and these have been investigated both in humans and nonhuman primates. In humans, deleterious social relationships, such as those that occur in the context of abuse, have similar negative outcomes on physical health and mental health (for a review see: Springer et al., 2003)—the experience of abuse greatly increases the prevalence and progression of disease. The quality of social relationships also plays a role in shaping health outcomes across the lifespan. Patterns of these effects have largely been elucidated in nonhuman animals. At one extreme, nonhuman primates raised in social isolation develop behavioral pathologies characterized by an inability to regulate emotion-related behavior and experience (e.g., Harlow et al., 1965; Mitchell et al., 1966). The pathology resulting from limited social contact is so extreme that one interpretation is that it induces clinical depression (Harlow and Suomi, 1974). In addition, natural or experimentally induced variation in the quality of maternal care impacts neuroendocrine, immune, and neurotransmitter system activity, and these differences are detected into adulthood (Coplan et al., 1996; Levine and Mody, 2003; Vandeleest et al., 2013). Similarly, social stress as related to dominance rank in nonhuman primates is related to a variety of physiological outcomes including reduced synaptic plasticity and dendritic atrophy, immunosuppression, reduced gonadal hormones, and pathogenic cholesterol profiles and hypertension (for a review see: Sapolsky, 2005). Finally, social instability (i.e., new social group formation or frequently rotating small group membership alters neuroendocrine function and sympathetic innervation of lymphoid tissue, and reduces survival after infection with a HIV-like virus; Mendoza et al., 2001; Sloan et al., 2007; Cole et al., 2009). In contrast to these negative effects is the growing evidence that both humans and nonhuman primates that have many social connections are buffered against stressful experiences (Berkman, 1984), experience less loneliness (Cacioppo et al., 2002), recover more fully from acute episodes of depression (George et al., 1989; Corrigan and Phelan, 2004), experience less disease (Seeman, 1996) and live longer (Steptoe et al., 2013).

Studies of nonhuman primates demonstrate that individual-level factors (e.g., personality, genetic predispositions) moderate the effects of social factors on health. For example, although social instability has been associated with negative health outcomes, the magnitude of impact depends on the personality of the individual. Under experimentally-induced social instability (rotating membership of small groups), rhesus macaques rated to be less “sociable” in adjective-based personality assessments had elevated plasma cortisol concentrations and poorer immune responses to an HIV-like virus when compared to animals that were more sociable (Capitanio et al., 1999, 2008). Similarly, a polymorphism in serotonin transporter gene promoter region (5-HTTLPR) has been shown to moderate the effects of impactful momentary social experiences. Peer-reared infant rhesus macaques with a short allele of 5-HTTLPR showed higher adrenocorticotropic hormone level in response to separation compared to those who were mother-reared or those with long alleles of 5-HTTLPR (Barr et al., 2004). Individuals carrying the short alleles of 5-HTTLPR had greater depressive symptoms when experienced greater early or recent adversities, but fewer depressive symptoms when adversities were absent or experienced supportive environments (Taylor et al., 2006). Together these examples highlight the need for complex dynamic modeling techniques that allow for the simultaneous modeling of complex social systems and their interaction with individual characteristics (e.g., personality or genotype) to fully understand their impact on health outcomes.

The mechanisms by which the effects of social life manifest as predictors of individual health outcomes is not clear and have been, to date, largely speculative (House et al., 1988; Adler et al., 1994). One reason for this speculation, rather than the specification of causal models, is that both human and nonhuman primate research to-date has been largely limited to small assortments of specific features of social processes (e.g., number of social connections, the general social class to which an individual belongs, the ordinal or cardinal rank of the individual in the group, or the impact of specific social relationships; Christakis and Fowler, 2007; Fowler and Christakis, 2008; Lewis et al., 2012). Such approaches ignore the multiscale and temporally dynamic structure of social life—that is that individual relationships differ in number, quality and type that are embedded within multiple broader social contexts of family, social class, and community. Further, individuals can belong to multiple groups that differentially overlap and which may exert differential influence on the individual at different times or in different contexts. Factors like these shape an individual's “social role” and are known to relate to both physical and mental health (Phillips, 1981; Murrell et al., 1992; Brissette et al., 2002; Litwin and Shiovitz-Exra, 2010) and yet are incredibly challenging to model in humans. A solution to these issues is to use a nonhuman primate model of human social-life and health in concert with sophisticated novel statistical approaches.

We believe that the solution to modeling the complexity of social life as it impacts health is to adopt a quantitative method that use a *multi-scale, dynamic* approach. Multi-scale refers to integration of information about the individual, his or her primary social relationships, larger social group, and society in concert with the individual's biological systems (which are also multi-scale). *Dynamic* refers to the ability to quantify both slow and rapid frequency changes in the variables of interest. Such an approach to human health might seem only plausible through the study of humans. However, a large and growing body of evidence from nonhuman primates, and specifically the genus *Macaca*, suggests that our primate cousins exhibit sufficient complexity and variability in social dynamics and similar links between social dynamics and health to be good models for humans (Flack et al., 2005; Flack and de Waal, 2007; McCowan et al., 2008, 2011; Kutsukake, 2009; Lehmann and Ross, 2011; Beisner et al., 2011a,b, 2012; Daniels et al., 2012; Dobson, 2012; Micheletta et al., 2013; Evers et al., 2014). Modeling the effects of the social environment using a systems science approach on a nonhuman primate model is not only increasingly feasible but may be superior to less controlled human studies, with the potential to uncover significant biomedical breakthroughs that standard non-computational approaches lack in both scope and depth.

## Nonhuman primates as an ideal model system

New computational techniques (described below) represent incredibly powerful tools that can be used to understand the relationships between social and biological systems. These tools require appropriate and robust network data and health data from large samples. While human research provides one potential avenue for such research, nonhuman primate research provides a unique opportunity to obtain intensive social network and health data across time. Complex study designs involving multiple scales and dynamic relationships over the lifespan are difficult to conduct on human populations for multiple reasons. First, it is nearly impossible to collect multi-generational data over the time course of a single study. Monkeys, however, have substantially shorter lifespans and faster life histories—macaques reach sexual maturity at ~4 years of age. Second, true population modeling of social network dynamics in humans is difficult because it is nearly impossible to define all participant nodes in the network. For every person in the network, there are thousands of others they do not know who may be able to influence their behavior or health via indirect connections. In contrast, every individual in the network is known and can be observed in groups of captive nonhuman primates. Further, the data used to generate networks are collected by direct observation rather than self-report, which is subject to recall bias and error.

In addition to being able to face the challenges posed by human research head on, nonhuman primate research offers some additional benefits. These benefits are particularly true of studies conducted with nonhuman primates living in large outdoor social groups. The genetic, biobehavioral, and social history of each macaque and social group can be fully characterized. Communities are sufficiently large (yet readily observable) to generate social heterogeneity at individual, family, and community levels. Nonhuman primate communities can be systematically perturbed (within ethical bounds) to elucidate causal links between social relationships and health. Finally, macaque communities are housed in controlled but complex environments but vary with respect to individual attributes and family and community structure across social groups.

Considering the environmental and social complexity is critical when selecting a model system and organism for human health. Most biomedical studies with nonhuman primates involve either indoor singly or pair-housed subjects or indoor/outdoor small-group housed subjects (Baker, 2007). Yet, research into the importance of the social environment on health requires greater environmental and social heterogeneity that mirrors human experience. Studies of nonhuman primates in large social groups offer just that possibility.

## Defining the new approach and its value

Recent computational and statistical developments allow for the development of new tools to evaluate the multi-scale and dynamic aspects of social life. Contemporary network science is one field that has expanded its toolkit dramatically in the wake of these recent developments. Although network science is most commonly understood in the context of *social* networks, social network analysis is one component of a much broader set of network science approaches that are all designed to reveal emergent structure in any type of system. Network science therefore has significant potential to provide an important set of tools that allow us to move beyond simple associations toward more predictive and causal models. The power of these predictive models can then be harnessed to develop specific, even “individualized,” interventions to promote human health, such as those sought after in the current goal to achieve “precision medicine.”

**Figure 1:**
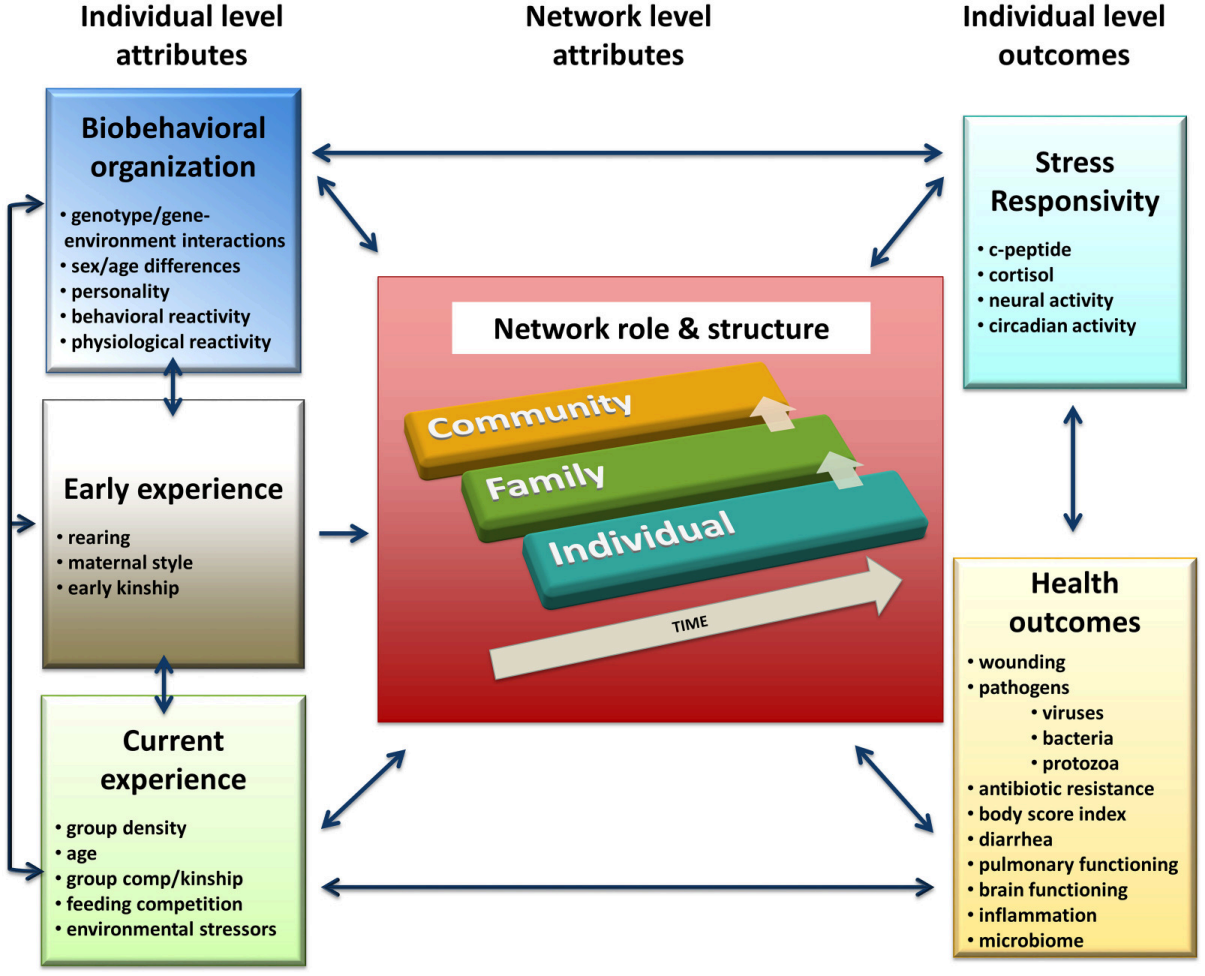
**Unifying conceptual framework for effects of individual characteristics and environmental/social stressors on network structure and dynamics leading to positive or negative health outcomes at each life stage and across the lifespan**. Arrows indicate possible direction(s) of effects including reciprocal relationships.

Developing predictive, causal models requires basic information about how the spatial and mathematical relations of networks relate to the content and quality of relationships at the individual, family, and community levels. Further, it is critical to understand how variation in these relationships at different levels influences health and health-related outcomes both in the short- and long-term. Network structure must also be linked to the fundamental characteristics of the individuals in the network and to the environmental and social contexts in which individuals interact, and this is in both the past and present. Utilizing such an approach will provide greater insight into how and why basic behavioral and social processes influence specific health outcomes as well as overall health and well-being (see Figure 1).

## Innovation in methodology: computational network analysis

Over the past few years our group and other research groups studying social networks have made significant strides toward developing innovative techniques for analyzing complex network structures that exhibit multiple spatial scales (e.g., individual, family, community levels), multidimensional topology (e.g., complexly correlated networks of different types of behaviors or relationships—such as friendship, Facebook and book club networks), and longitudinal temporal dynamics (e.g., changes to friendship networks over time; Berkman, 1984; George et al., 1989; Seeman, 1996; Cacioppo et al., 2002; Corrigan and Phelan, 2004; Capitanio et al., 2008; Steptoe et al., 2013). These are bottom-up (data-driven) model-generating approaches have been developed with biological and social data in mind and have been tested on these types of data. Importantly, these new techniques have few underlying assumptions and seek to understand the underlying mechanisms producing patterns in relationships. They are designed to examine the hierarchical and dynamic architecture of networks, such as connectivity and community structure, information flow through networks, and joint relationships between networks. Two key aspects of network structure need to be modeled to address complexity in network structures: (1) multi-scale spatial structure (e.g., differences in network structure at different scales) and (2) multi-scale temporal dynamics (e.g., changes in network structure across time). Additionally, networks differ in the major characteristics and geometry that define them. Data represented by a single (one-mode) network (e.g., friendship network) and data represented by two or more networks (two-mode or bipartite networks) juxtaposed to one another (e.g., Facebook network and book club membership network considered together) have different geometric structures. In each geometric structure, networks can differ in the characteristics that define them. Networks can be comprised of either presence or absence of the relationship (e.g., Susan and Peter are Sam's Facebook friends, which is binary, or unweighted) or weighted by the number of interactions (e.g., Sally texts Sam five times per day). Each type of network also can consist of either undirected links or ties (Sally and Sam are friends on Facebook but no information is known about who “friended” whom or who initiates communication with whom) or directed links or ties (Sally walks toward Sam often but Sam walks away when she nears). As such, the appropriate techniques for modeling a binary, undirected network are very different from those necessary for a weighted, directed network. As an example of this growing area of computational development, below we describe some of our new models that address the analysis of these different network types as well their further development below. These methods work on social network data where individuals (nodes) are connected via behaviors that are either directed (i.e., each edge is drawn from a source individual to the receiving individual) or undirected (i.e., edges are shared without direction) and either weighted (i.e., edges are defined by the rate or frequency of interactions) or unweighted (i.e., edges are defined by presence or absence of an interaction). See Table 1 for a summary.

**Table 1:** **Summary of computational network approaches**.

**New computation method**	**Purpose**	**Directed edges**	**Weighted edges**	**Description of output**
Percolation and conductance	Method for quantifying how information flows through directed networks via direct and indirect pathways	Yes	Yes	Identifies all direct and indirect pathways in a network. Summarizes the overall consistency in the direction of these pathways for every pair of nodes (e.g., 100% of 1-step paths and 90% of 2-step paths flow from A to B), yielding a measure of the probability that any path flows from A to B vs. B to A.
Joint modeling	Method for modeling the inter-dependent relationship across multiple networks	Yes	No	Categorizes each pair of nodes according to their joint-network relationship (using a vector). Identifies a model whose predictors describe what drives the observed frequencies of each type of pair, such as abundance of pairs with opposite direction phone calling and e-mailing.
Data cloud geometry	Clustering methodology that identifies a community structure via a data-driven random walk	No	Yes	Identifies block structure in a matrix by clustering together nodes that are most similar. Creates a hierarchical tree that describes the multi-scale structure of the network.
Data mechanics	Method that allows the discovery of clusters of nodes from one network that are grouped based upon similarity across a series of metrics	Both directed and undirected	Both weighted and unweighted	Identifies block structure in a bipartite matrix by shuffling the ordering of rows and columns, yielding two hierarchical trees (one for rows, one for columns) that describe the inter-dependent relationship between them.

### Percolation and conductance

**Figure 2:**
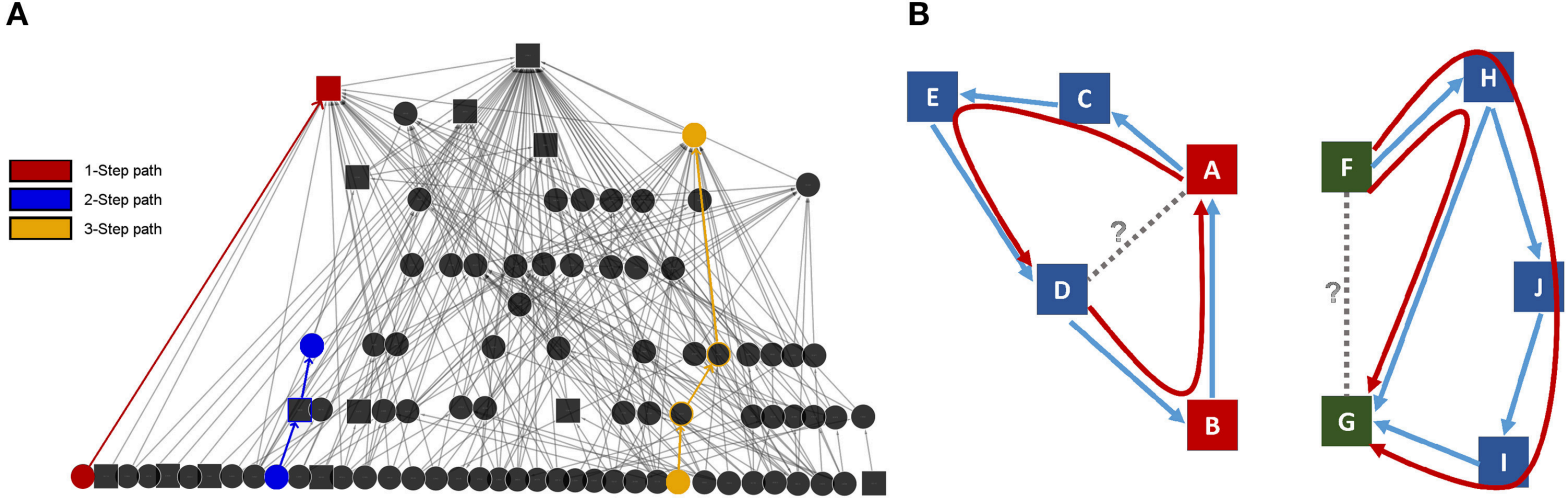
**Percolation and Conductance uses indirect pathways to determine consistency in a network. (A)** Behavioral network illustrating direct (blue) and indirect pathways (yellow, red). **(B)** Low consistency from A to D represented by green and red arrow indicates an ambiguous dominance relationship. The high consistency from F to G represented by green arrows indicates high certainty that F outranks G.

Percolation and Conductance utilizes the information about both direct and indirect relationship pathways to characterize how information flows through directed networks (Fushing et al., 2011; Fujii et al., 2013; Table 1; Figure 2). This method uses a percolation algorithm to gather information from multi-step pathways network, which allows tracking and computation of the flow of information through indirect social interactions (e.g., Sally interacts with Sam who earlier interacted with John; Fujii et al., 2013). A conductance algorithm is applied to ensure that all potential flow pathways are explored. The contribution of each path to the imputed matrix is weighted by its likelihood of being successfully traversed during the random walk.

### Joint modeling

In a complex system, different social and biological networks rarely operate in isolation. Rather, dynamic relationships between different social behaviors often covary in highly complex, synergistic or even emergent ways. We would also argue that many negative and positive health outcomes likely have similar complexity in their etiology. Instead of analyzing these networks individually, we have developed methodology for analyzing networks at the same time (hence, “joint modeling”) which produces metrics that characterize the multi-system dynamics as a whole.

**Figure 3:**
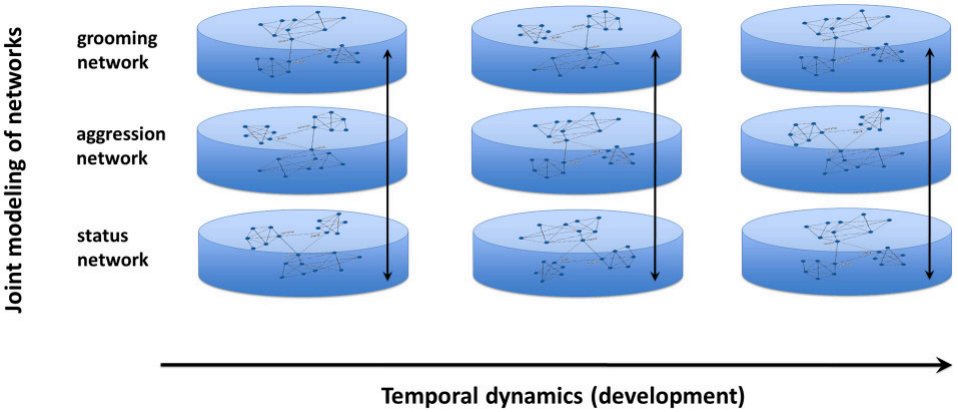
**Conceptual framework behind joint modeling of networks**. Using this new technique, multiple networks can be evaluated for how they correspond to one another and how that correspondence changes over time, across development, during the aging process, or over the lifespan.

Joint modeling uses a bottom-up iterative modeling approach (Chan et al., 2013) that begins with multiple component networks of the system (generated from empirical data) and links the networks via common nodes in each network (Table 1; Figure 3). The raw data are used to calculate expected probabilities of jointly observing a link in each network for every pair of individuals. For example, consider the e-mail and phone calling networks of a community of friends—joint network modeling begins by calculating what proportion of pairwise relationships are characterized by each possible combination of connections in the two networks, such as unidirectional e-mailing with bidirectional phone calling (e.g., Hank e-mails and calls Jane, but Jane only calls Hank and never e-mails him), or the absence of both e-mailing and calling interactions (e.g., John and Sally have never communicated with each other over e-mail or phone). In the null model, the relationships across interactions are assumed to be independent, e.g., knowing that John never calls Sally is independent of whether Sally and John e-mail each other. Constraint functions are then iteratively applied to tune these probabilities to match the observed directed network data.

Once appropriate constraint functions (based upon existing theory or data) are applied to match the observed data, the relationships between these networks then can be assessed over time. This interactive process allows one to examine how multiple networks correspond to one another at a given point in time or how the relationship of these networks changes longitudinally (Chan et al., 2013). Monitoring the complex dynamics of a system provides a valuable way of quantitatively characterizing the potential impact of an individual's social environment.

### Data cloud geometry

**Figure 4:**
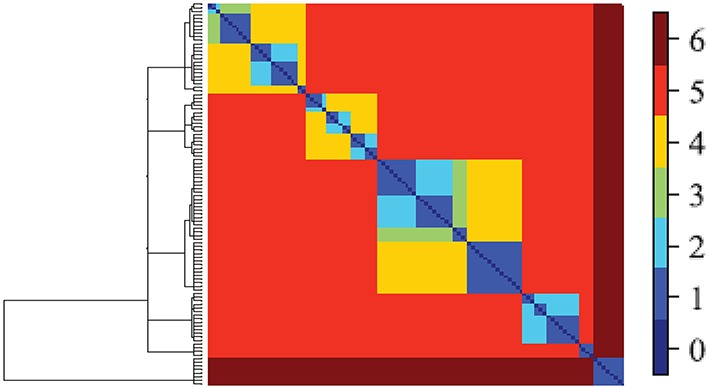
**Illustrative DCG-tree based on fMRI data (Fushing et al., 2013)**. Tree on left panel also corresponds to pattern on top panel. Note that different levels of the tree (known as “temperature”) yield different blocking patterns corresponding to communities. At the highest temperature indicated in dark red, a single community is observed; at the lowest temperature, 19 communities are observed. DCG finds the best clustering solutions at multiple levels in the tree.

All social systems have inherent multi-scale structure (e.g., individual, family, community). This multi-scale geometry must be quantitatively defined and preserved during the modeling process in order to model how individuals relate to each other and to health outcomes. Data cloud geometry (DCG) is a new method that identifies community structure at multiple scales by performing a random walk through an empirical network. A random walk creates a path by moving through the data step by step. The direction of each step is determined randomly and the probability of each step is guided by the data. Cumulatively, these random walks produce a similarity matrix describing how similar two nodes (e.g., individuals) are in their social connections. This similarity matrix captures the geometric structure of the network by deriving a hierarchy of clustering. Individuals with greater similarity in social connections are considered to be “closer,” and thus cluster together at a lower level of the hierarchical tree than individuals with few similarities in their connections (Table 1; Figure 4).

A key advantage of using DCG over other clustering algorithms is that DCG generates more accurate cluster membership than previous approaches because outliers do not, by default, cluster with each other (i.e., a cluster of outliers) simply because they significantly differ from other clusters (but may not be similar to one another). Instead outlier individuals must be similar to each other to be in the same cluster. Second, this approach has a built-in mechanism for self-correcting clustering membership across different levels, which is important for inferences of cluster membership across different levels of analysis (e.g., clusters individuals at family level in comparison to clusters of families at community level). These two features allow robust classification of data into groups and thus identify community structure at multiple levels of analysis (e.g., how individuals group within families, and families into communities).

### Data mechanics

**Figure 5:**
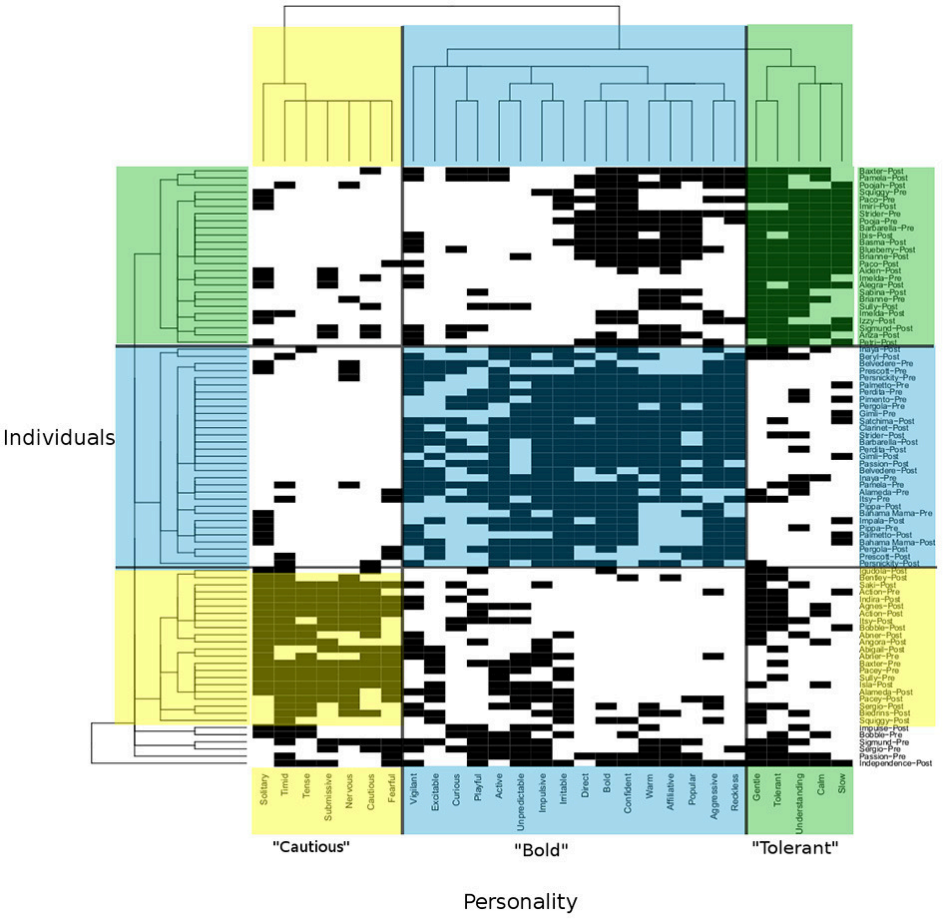
**Data Mechanics tree and heat map representing bipartite network analysis of clusters of individuals (macaques) set on the Y-axis with clusters of personality type on the X-axis**. Note the blocks of individuals sharing personality profiles on the Y-axis as well as the blocks of personality traits on the X- axis. For illustration for the level in the tree where the red line is located, we can block personality according to individuals and individuals according to personality (highlighted yellow, blue, and green boxes). Note how individuals who are similar in personality profiles group together and personality traits corresponding to personality types group together.

Data Mechanics begins with a bipartite matrix in which the rows represent a set of individuals from a network or system and the columns represent various metrics for which each individual has been assessed (Table 1; Figure 5). The rows and columns are then shuffled iteratively to reveal blocks of similar subjects. Shuffling occurs using principles from thermodynamics to assign “energy” to the organization of the network matrix. Network matrices with the lowest energy state are selected as the “best fit” for these blocks of subjects. Notably, this method generates a hierarchical tree of profiles that allows for the identification of higher order behavioral and social profiles that relate to negative (or positive) health outcomes in predictive or causal ways. Data mechanics (Fushing and Chen, 2014) can be used to identify clusters of individuals that share similar health profiles and social profiles as well as determine their inter-relationships over time. This new technique will allow us to quantify the collective influence of one's social environment on aspects of individuals' health status.

## An example: computational network applied to a nonhuman primate model as translation for humans

Below we discuss our group's work to provide brief, preliminary examples of the application of our computational network approach to a nonhuman primate social system as a translational model for enhancing human health. We focus on two examples. In the first example, we illustrate the effects of social network structure on individual health at a single time scale. In the second example, we illustrate the effects of social network on group health at multiple time scales (which in turn can positively or negatively influence individual health across these temporal scales) to illustrate the multi-scale (e.g., individual, group), multidimensional (e.g., joint modeling) and longitudinal aspects (before and after perturbation) of our holistic systems approach.

### Example #1: predictability of status as predictors of individual heath

Indicators of social rank in humans, such as socioeconomic status, are thought to be a source of stress. Yet studies on the presence and direction of this relationship between rank and stress in animal societies have been quite equivocal in terms of whether it impacts health. Preliminary results from our research group using computational network models, as outlined above, suggest that certainty or ambiguity of one's dominance relationships may have a greater impact on health than actual rank itself. Certain dominance relationships are those in which it is very clear who is dominant and who is submissive. In contrast, ambiguous dominance relationships are those in which the identity of the dominant and submissive individual is not clear. We can quantify the number of these relationships per individual into a metric that reflects an individual's general propensity to have dominance interactions that go with the global network “flow” of dominance (i.e., pathways in the network generally flow from dominance to subordinate individuals) or interactions that run counter to the global flow of dominance (what we term “dominance certainty”).

We used “Percolation and Conductance” (Fushing et al., 2011; Fujii et al., 2013; see Section Innovation in Methodology: Computational Network Analysis above), to quantify dominance certainty. The dominance relationship of each dyad in the group is quantified using both direct and indirect agonistic interactions, which yields a holistic quantitative measure of the degree of certainty vs. ambiguity of dyadic relationships in the network.

Dominance certainty relates to health outcomes in situations where dominance rank does not. First, preliminary analyses of both physiological parameters as well as physical manifestations of stress (e.g., blood cytokine levels estimating inflammatory responses, frequency of diarrhea bouts, frequency of trauma, and severity of trauma) demonstrate that having ambiguous dominance relationships exhibits a dose dependent relationship with greater incidence of diarrhea and trauma, and higher cytokine levels (Vandeleest et al., 2014a,b; Beisner et al., 2015). Individuals with lower dominance certainty have higher incidences of poor health. Additionally, for the pro-inflammatory cytokines, dominance certainty modified the effect of dominance rank such that individuals with lower dominance certainty only experienced poorer health if they were also high-ranking. In other words, having ambiguous dominance relationships only negatively impacted cytokine levels for individuals that stood to lose their high status position (i.e., low-ranking individuals with ambiguous relationships stand to *gain* status). This suggests that uncertainty in relationships, such as dominance, may be more important for health outcomes than actual dominance status. Second, cohesion or interconnectedness in affiliative relationships, such as grooming, also may have an effect on health both directly and indirectly through dominance certainty. An analysis of community membership in a grooming network, using “Data Cloud Geometry” (Fushing and McAssey, 2010; Chen and Fushing, 2012; Chan et al., 2013; see Section Innovation in Methodology: Computational Network Analysis above), demonstrated that subjects who shared a grooming cluster with more family members evidenced less variance in their dominance certainty when compared to subjects who shared a grooming cluster with few family members. Thus, cohesion in affiliative relationships may influence certainty in relationships outside the affiliation context. Finally, personality is also associated with dominance certainty. Rhesus monkeys who score high in the trait “confidence” and who are also lower-ranking, have more ambiguous dominance relationships than lower-ranking individuals who are less “confident.” It is possible, even probable, that confident individuals may challenge dominance relationships more frequently than animals who are not confident.

Taken together, these results demonstrate that family or community cohesion and certainty in relationships interact with properties of the individual, such as personality, to have a significant impact on aspects of health. Notably, these preliminary analyses indicate that certainty about one's position in social relationships may be more important than absolute rank in predicting health outcomes. We can determine thresholds for how much uncertainty or how many uncertain relationships, and with whom, are sufficient to impact health. However, distinguishing between actual class and predictability in relationships are likely confounded in many human societies. Therefore, these results from a nonhuman primate model can inform how measurement of actual direct and indirect social relationships, rather than simple socioeconomic class designations, can impact human health through a number of biological systems.

Indeed, the evidence from this work clearly indicates that knowledge beyond direct relationships, that is how individuals fit within the global social structure (i.e., fit with the global flow of dominance; fit with family-related clustering structure), is critical to understanding health. No longer is it enough to measure what an individual's direct social interactions are (e.g., number of friends, etc.); rather we need to know more precisely how those interactions are embedded within the structure/geometry of their social community.

### Example #2: stability of the social environment and group health

The stability of human and nonhuman social groups impacts health (e.g., German and Latkin, 2012). Animal societies are complex behavioral systems in which the dynamics of the system as a whole represent the synergistic interaction among multiple behavioral networks. Stability is an emergent property of the interactions between networks (e.g., Barrett et al., 2002). Ideally, then, the information from these separate behavioral networks should be combined in order to achieve a comprehensive understanding of a given system's stability (Barrett et al., 2012; Hsieh et al., 2014). This can now be accomplished with “Joint Modeling” (see Section Innovation in Methodology: Computational Network Analysis above).

Assessing the joint relationship between two behavior networks, such as aggression and status signaling in rhesus macaques, across multiple time points can reveal whether (a) the two networks interact in a predictable manner and (b) whether the pattern of interdependence between the two networks changes over time, particularly in response to perturbations to the system. In a recent study, we constructed two unweighted networks (aggression and status) for seven large outdoor captive groups of rhesus macaques. The groups varied in their stability—four groups were stable, three groups had to be disbanded completely after our data collection ended as the result of severe and widespread trauma. Macaque society is organized primarily by dominance relationships (e.g., Sade, 1967, 1969). In a society with a clear dominance hierarchy, most pairs of individuals are expected to have clearly communicated dominance relationships. We expected that the joint relationship between aggressive interactions (e.g., threatening, biting) and status interactions (e.g., facial expression to signal formal acceptance of subordinate status—the silent bared teeth display) should, in general, agree with the known pattern of dominance. Aggression and status are directed networks in which one monkey in a given dyad (the initiator) either aggresses or signals to the second monkey in a given dyad (the recipient; Chan et al., 2013; see Section Innovation in Methodology: Computational Network Analysis above).

Joint Modeling revealed that stable groups all showed the same pattern of interdependence between aggression and status networks—in all groups, far more pairs than expected had relationships involving unidirectional aggression (from dominant to subordinate) along with unidirectional status signaling (from subordinate to dominant), yet other potentially problematic types of relationships (e.g., bidirectional aggression with no status signaling) occurred as frequently as expected by chance (Beisner et al., 2015). In stable groups, a highly complex dependent relationship between the aggression network and status network is present across time. That is there was a strong association between aggression is one direction and status signaling in the opposite direction. In contrast, the three unstable study groups exhibited a change in their pattern of network interdependence (Beisner et al., 2014) prior to the onset of extreme aggression. Two of the three unstable groups showed a dramatic reduction in the extent of interdependence between aggression and status networks. In other words, knowing that monkey A threatens monkey B does not necessarily mean that monkey B will signal its subordinance to A, as in stable groups. Thus, loss of interdependence between two behavioral networks is one feature of social instability. Network data can therefore be used to predict whether a group is at risk of social collapse (Hsieh et al., 2014). Critically, this same pattern of effects was observed in the banking industry prior to the 2008 financial crash—suggesting that patterns of change in joint models are translationally relevant.

All social systems are composed of multiple interconnected networks, but until now, it has been impossible to evaluate them simultaneously. Joint Modeling may be of greatest utility in quantifying the impact of environmental, ecological, or social changes over time on the underlying structure of a social group and their consequent health outcomes. Further analyses are underway to assess these effects on such health outcomes across the lifespan at the group, family and individual levels in our populations of rhesus macaques.

## Conclusions and future research

The computational network approach used on data collected from large outdoor social groups of nonhuman primates promises a broad translational tool that can realistically model individual, family, and group health across the lifespan in human populations. The approach and examples we described here, however, are only the beginning. While nonhuman primates have served as a translational model for humans for decades, the importance of the complexity of the social and physical environment in biomedical applications is still essentially unrealized (e.g., Capitanio and Emborg, 2008; Shively and Clarkson, 2009). Future biomedical research should address this issue directly by using socially and environmentally relevant subjects whenever possible. Indeed, with newer more advanced and less invasive methods for collecting and analyzing biological samples in the field, wild populations of nonhuman primates may provide additional translational opportunities. Field-based biomedical research on genetically, socially, and behaviorally well-characterized nonhuman primate populations could transform our understanding of threshold, collective, and emergent effects of the social environment on health outcomes.

In contrast to the nonhuman primate as a biomedical model, computational approaches for modeling multidimensional and dynamic systems are only in their infancy, but the computational power and intellectual drive to model complexity in spatial and temporal dynamics is certainly now present. Further development of this approach could revolutionize medicine by allowing us to develop individualized medicine on an unprecedented scale. We believe that this innovative systems science approach for studying complex emergent health outcomes on socially and environmentally complex nonhuman primate models in either captivity or the field promises better and more timely solutions to the greatest physical and mental health challenges we currently and will continue to face as humans.

## Author contributions

BM: Developed conceptual framework, funded research, wrote manuscript. BB: aided in writing of manuscript. JV: aided in writing of manuscript. EBM: aided in writing of manuscript. JJ: aided in writing of manuscript. DH: aided in writing of manuscript. FH: developed computational tools.

### Conflict of interest statement

The authors declare that the research was conducted in the absence of any commercial or financial relationships that could be construed as a potential conflict of interest.
